# A Feasibility Study of Processing Polydimethylsiloxane–Sodium Carboxymethylcellulose Composites by a Low-Cost Fused Deposition Modeling 3D Printer

**DOI:** 10.3390/ma11091578

**Published:** 2018-09-01

**Authors:** Paola Calcagnile, Gabriele Cacciatore, Christian Demitri, Francesco Montagna, Carola Esposito Corcione

**Affiliations:** Dipartimento di Ingegneria dell’Innovazione, Università del Salento, via Monteroni, Km 1, 73100 Lecce, Italy; paola.calcagnile@unisalento.it (P.C.); gabriele88cacciatore@gmail.com (G.C.); christian.demitri@unisalento.it (C.D.); francesco.montagna@unisalento.it (F.M.)

**Keywords:** 3D printing, polydimethylsiloxane, sodium carboxymethylcellulose, rheology, biomedical applications

## Abstract

Additive manufacturing (AM) techniques allow the construction of complex physical models reproducing the content of a specific CAD file, and, among them, Fused Deposition Molding (FDM) stands out for its many advantages. The aim of the present work is to perform a feasibility study of 3D printing of a model of human heart to be used to simulate surgical operations or for training through a two-step method based on extrusion and FDM processes. To this purpose, typical extrusion instrumentation and a simple and low-cost FDM printer are employed, in combination with a thermoplastic polydimethylsiloxane (PDMS), chosen for its transparency, flexibility, and high resistance to multiple agents and aging. To improve its tactile properties and mimic the slimy effect of living organs, sodium carboxymethylcellulose (Na–CMC) fibrils are added to it. The starting materials, the neat PDMS filament and the composite one, are deeply characterized in terms of structural, thermal, and rheological properties in order to fix the most suitable extrusion and FDM parameters. The composite filaments show larger diameter and roughness, which cause undesirable effects during 3D printing, such as episodic nozzle obstruction, and exhibit a faster degradation, making the FDM step difficult. Nevertheless, the major issues are related to the low crystallinity degree of the employed polymer. The feasibility study carried out leads to the printing of composite layers, even though far from the desired final target. Possible solutions to print the fully characterized Na–CMC/PDMS composite are addressed in the conclusion of this work.

## 1. Introduction

According to the International Organization for Standardization (ISO)/American Society for Testing and Materials, additive manufacturing (AM), commonly referred to as “3D printing”, is defined as the “process of joining materials to make parts from 3D model data, usually layer upon layer, as opposed to subtractive manufacturing and formative manufacturing methodologies” [1]. In AM, the part is built “layer by layer”, reproducing a 3D CAD model, with the major advantage that adjustments in the produced parts may be quickly introduced by modifying the electronic design file [2,3]. AM has emerged as a cost-effective, efficient, and customized manufacturing option in countless applications, moving from automotive to design and art and even space, as a 3D printer was successfully employed by scientists to produce parts and perform experiments during the NASA mission concluded in September 2017 [4].

AM is successfully used for the production of customized medical devices, such as dental implants, hearing aids, prostheses, surgical instrumentations, models for students, or for professionals to study and plan appropriate surgical strategies [5,6,7]. Moreover, the integration of computer-aided design, advanced imaging techniques, and rapid prototyping has permitted the fabrication of objects with both macro- and microscale control, customized for every single patient [8,9,10,11].

Among AM technologies, Fused Deposition Modeling (FDM) is one of the most popular. In FDM, a thermoplastic polymer in the form of a filament rolled up in a spool is drawn through a nozzle, which heats up and applies shear forces to the filament, thus melting it and decreasing its viscosity. In this way, polymer layers are subsequently deposited one on the other, bonding and hardening instantaneously [3,12].

FDM exhibits some relevant advantages, as (1) materials can be handled and processed with great ease and flexibility, (2) materials’ residence time in the heating step is very short, and (3) it is possible to perform a continuous production process [13].

In recent years, developments in biocompatible materials research have enabled 3D bioprinting of models of functional living tissues for applications in regenerative medicine. For instance, Corcione et al. [6] recently investigated the potential of FDM to produce an osteogenic hydroxyapatite–polylactic acid bone-graft substitute.

Other research groups have employed 3D printers to fabricate a knee meniscus, heart valve, spinal disk, other cartilages and bones, and even an artificial ear [14,15,16]. Wang et al. [17] used 3D bioprinting to deposit cells within different biocompatible hydrogels to create an artificial liver. Bartlett [16] reports the use of a 3D printer combined with CT images of a patient’s airway allowed them to produce a precisely modeled, bioresorbable tracheal splint that was successfully implanted. Extrusion is a fundamental method to produce a filament suitable for FDM. In extrusion, high temperatures and shear rates are applied to a low-viscosity thermoplastic polymer in order to render it even less viscous and able to flow easily. Thermoplastic materials typically used in the extrusion process include polylactic acid (PLA), acrylonitrile–butadiene–styrene (ABS), and polystyrene (PS) [3]. The present work represents a feasibility study of 3D printing of a model of human heart to be used for training and simulating surgical operations through a two-step approach based on consecutive extrusion and FDM processes, by means of a typical extrusion instrumentation and a simple and low-cost FDM printer. To this purpose, a silicon-based thermoplastic polymer is selected, as characterized by transparency, high resistance to chemical and corrosion attack, to temperature, and aging. It also has the proper flexibility and nonsticky character suited to reproduce living organs. In order to mimic the tactile properties of human heart, organic cellulose-based fibrils are employed as a filler in the polydimethylsiloxane (PDMS) matrix. Primarily, the biocompatible and biodegradable composite material is obtained via a solvent-free process, with a maximum loading capacity with filler of 10% in weight. It is then subjected to the extrusion step in order to obtain a regular filament that is subsequently processed by the low-cost FDM printer to build the desired object.

First of all, the physical and chemical properties of the starting materials are analyzed. Subsequently, the rheological properties of the starting materials and of the bare and composite polymer in the form of a filament are characterized to set the most suitable extrusion and FDM-process parameters. Although the performed printing process permits to obtain well-adherent layers of composite material, it is noticed that irregular samples are obtained, thus indicating that the employed combination of materials and a low-cost 3D printer did not allow a fine control of geometry.

## 2. Materials and Methods

### 2.1. Materials

Pellets of thermoplastic Geniomer^®^ 145 PDMS, supplied by Wacker Chemie AG (Munich, Germany), containing over 90% siloxane were used as the incipient material. The filament for the 3D-printing process was obtained by melt extrusion with a twin-screw extruder (Haake Reomix 600/610, Rezzato BS, Italy), using PDMS only, or adding 10 mass% of food-grade Aqualon^®^ Na–CMC, high viscosity, minimum purity 99.5% (7H4F 9000LPS, Ashland, Covington, KY, USA), in order to improve the tactile properties of the final printed product.

### 2.2. Preparation of PDMS and PDMS-Based Composite Filaments

Filaments suitable for 3D printing have been obtained by melt extrusion with a (Haake reomix 600/610) twin-screw extruder, starting from PDMS and Na–CMC. The extruder is divided into six zones of 60 mm of length that can work at different temperatures. The L/D extruder ratio is constant and equal to 25, while the screw diameter is 16 mm. A soon as the material exits from the circular die (with a diameter of 3 mm), it is water-cooled and coiled on a spool. In order to obtain a filament of 1.75 mm diameter (required value for the 3D printer) and to offset the polymer swelling at the exit of extrusion die, (1.8 mm diameter size), the distance between the die extruder and the water bath was optimized at 25 cm. The employed parameters have been fixed through the performed experiments and summarized in Table 1 of the Results section.

### 2.3. 3D Printing of PDMS and Na–CMC/PDMS Composites by FDM

A 3DPRN LAB 3D (TIPS, Castiglione M.R., TE, Italy) was employed with both neat and loaded filaments. A nozzle of 0.4 mm, a layer height equal to 0.1 mm, a speed of extrusion of 30 mm s^−1^, and temperature of 230 °C were set.

### 2.4. Characterization on PDMS

Fourier Transform Infrared Spectroscopy (FTIR) has been performed in order to clarify the composition of PDMS. Infrared spectrum has been recorded by a Perkin Elmer Spectrum One Fourier Transform spectrophotometer at 4 cm^−1^ resolution. The spectrum has been acquired in the wavenumber range 400–4000 cm^−1^, with 32 scans.

The crystallization of the PDMS has been monitored through X-ray Diffraction (XRD) analysis. The XRD (Rigaku, Tokyo, Japan) pattern has been acquired with Cu Kα radiation (λ = 0.15406 nm), focus size 0.4 mm × 12 mm, rated tube voltage 40 kV, goniometer radius 285 mm, and recorded in the region of 2 θ from 5° to 60°.

Thermogravimetric analysis (TGA) has been performed on a TGA-1 analyzer (Mettler-Toledo) in the temperature range 20–1000 °C, with a heating rate of 10 °C min^−1^ and under a nitrogen flux of 50 mL min^−1^, in order to determine the effective solid residue and the degradation temperatures of each component. The mass loss was normalized with respect to the initial dehumidified sample mass and reported as a function of temperature.

The rheological characterization of PDMS pellets has been performed by using a rotational rheometer (ARES, TA Instruments, New Castle, DE, USA) equipped with cone-and-plate geometry.

The viscoelastic behavior of PDMS pellets has been analyzed by isothermal dynamic rheological characterization, respectively at 190 °C (the temperature at the die) and 205 °C. We measured the variation of the complex viscosity (η*) as well as the frequency-dependent storage (G′) and loss (G″) modules. Dynamic time-sweep tests have been conducted at a constant strain of 1% with a frequency of 0.5 Hz as a function of the time. All the rheological experiments were repeated at least three times to check the repeatability of the results.

Finally, the trend of viscosity with respect to the shear rate, at 170, 190, and 205 °C was measured on the different filaments produced, in strain-control mode. A cone-and-plate configuration (radius = 12.5 mm) was used, and the shear rate ranged between 0.01 and 100 s^−1^.

As the rotation speed of the screw influences the rate at which the material comes out from the die, and consequently the dwell time at a given temperature inside the extruder, a dynamic rheological test was made with a heating rate of 5 °C/min from 100 °C (the temperature set in the feeding zone of the extruder) to a temperature of 205 °C (the central area of the extruder in which the maximum temperature is reached).

Isothermal characterization, respectively at 190 °C (the temperature at the die) and 205 °C, has been carried out by placing a sample of pellets on the surface of the plate of the rheometer and setting the gap of 1 mm.

### 2.5. Characterization of Na–CMC Powder

FTIR spectrum has been recorded by a Perkin Elmer Spectrum One Fourier Transform spectrophotometer at 4 cm^−1^ resolution. The spectrum has been acquired in the wavenumber range 400–4000 cm^−1^, with 32 scans.

The crystallization of Na–CMC powder has been monitored through XRD analysis. The XRD (Rigaku, Tokyo, Japan) pattern has been acquired with Cu Kα radiation (λ = 0.15406 nm), focus size 0.4 mm × 12 mm, rated tube voltage 40 kV, goniometer radius 285 mm, and recorded in the region of 2θ from 5° to 60°.

TGA has been performed on a TGA-1 analyzer (Mettler-Toledo) in the temperature range 20–1100 °C, with a heating rate of 10 °C min^−1^ and under a nitrogen flux of 50 mL min^−1^, in order to determine the effective solid residue and the degradation temperatures of each component. The mass loss was normalized with respect to the initial dehumidified sample mass and reported as a function of temperature.

The following Differential Scanning Calorimetry (DSC) program has been performed: heating scan in the range 0–190 °C, subsequent cooling scan in the range 190–45 °C, and further heating scan in the range 0–190 °C; all the steps performed at 10 °C min^−1^.

The structure of pure Na–CMC powder has been investigated by Scanning Electron Microscopy (SEM, Zeiss Evo 40, Oberkochen, Germany) equipped with energy-dispersive X-ray (EDX) spectroscopy system (Bruker x flash detector 5010, Billerica, MA, USA).

### 2.6. Characterization on Extruded PDMS Filaments

DSC analysis has been performed to compare the thermal behavior of the neat PDMS filament, cellulose powder, and the composite filament. For PDMS filament, the following process has been carried out: heating scan in the range 0–190 °C, subsequent cooling scan in the range 190–45 °C, and further heating scan in the range 0–190 °C; all the steps performed at 10 °C min^−1^.

TGA on PDMS filament has been carried out in the range 20–1000 °C, with a rate of 10 °C min^−1^, and under a nitrogen flux of 50 mL min^−1^.

Rheological properties of PDMS filaments have been characterized by means of an ARES rotational rheometer equipped with parallel plate geometry. All the other parameters were kept constant with respect to those used for the characterization of the pellets.

The morphology of pure PDMS filaments has been analyzed by Scanning Electron Microscopy (SEM, Zeiss Evo 40). The analysis was performed at different magnification values.

### 2.7. Characterization on Extruded Na–CMC/PDMS Composite Filaments

The following DSC program has been performed: heating scan in the range 0–190 °C, subsequent cooling scan in the range 190–45 °C, and further heating scan in the range 0–190 °C; all the steps performed at 10 °C min^−1^.

TGA on Na-CMC/PDMS composite filaments has been carried out in the range 20–1100 °C, with a rate of 10 °C min^−1^ and under a nitrogen flux of 50 mL min^−1^.

Rheological properties of Na–CMC/PDMS filaments have been characterized by means of an ARES rotational rheometer equipped with parallel plate geometry. All the other parameters were kept constant with respect to those used for the characterization of the pellets.

The morphology of the composite filaments and the distribution of Na–CMC powder in the polymer matrix have been analyzed by SEM (Zeiss Evo 40). The analysis was performed at different magnification values.

### 2.8. Characterization on 3D-Printed PDMS and Na–CMC/PDMS Samples by FDM

The morphology and superimposition of filament layers obtained by FDM 3D printing have been analyzed by SEM (Zeiss Evo 40). Images at different magnification were recorded.

## 3. Results and Discussion

### 3.1. Characterization on the Neat Starting Materials and the Composite One

**Figure 1:**
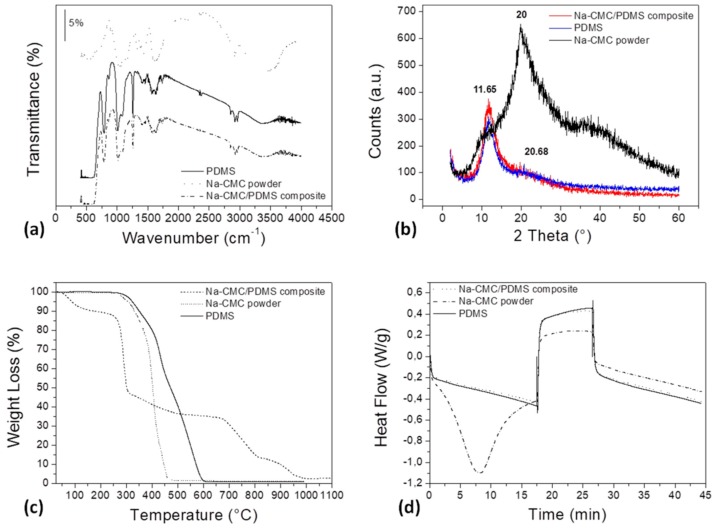
Comparison of the properties of neat polydimethylsiloxane (PDMS), sodium carboxymethylcellulose (Na–CMC) powder, and the Na-CMC/PDMS composite, in terms of: (**a**) Fourier Transform Infrared Spectroscopy (FTIR) spectra; (**b**) X-ray Diffraction (XRD) patterns; (**c**) thermogravimetric analysis (TGA) and (**d**) Differential Scanning Calorimetry (DSC) thermograms.

A full characterization of the neat starting materials, compared to the Na–CMC/PDMS composite, has been firstly performed and results are shown in Figure 1. Specifically, FTIR and XRD analysis have been carried out in order to investigate the chemical composition and structure of the employed materials, TGA and DSC thermograms were acquired to characterize their thermal properties, while rheological characterization of PDMS pellets has been performed as a preliminary step to the polymer-extrusion and 3D-printing processes.

In Figure 1a it is possible to notice the FTIR spectra of the different materials, while in Figure S1 of the Supporting Information file, the spectra of PDMS and cellulose powder, with their characteristic peaks, are shown in detail. The typical peaks of –CH_3_ symmetric rocking and Si–C symmetric stretching in (Si–CH_3_) lie in the range 789–796 cm^−1^. The asymmetrical (Si–O–Si) stretching vibrations are visible at about 1020–1074 cm^−1^, while the –CH_3_ symmetric bending in (Si–CH_3_) is evident at about 1260 cm^−1^ [18]. The peak at about 1411 cm^−1^ is ascribable to the –CH_3_ asymmetric bending in (Si–CH_3_), as confirmed in the literature [19]. The stretching vibrations of C=C and CH–(CH_2_) are also visible at 1632 cm^−1^ and 2866 cm^−1^, respectively. Then, the peaks at about 2801 and 2905 cm^−1^ are representative of the symmetrical and asymmetrical –CH_3_ stretching, respectively [19]. The presence of a broad peak at 3500 cm^−1^, typical of –OH functional group, is most probably due to adsorbed moisture (Figure S1a in Supporting Information file).

The characteristic peaks of the employed Na–CMC powder are: at about 1100 cm^−1^ the one typical of n(C–O) is identifiable, while at 1622 cm^−1^ the carboxyl groups n(COOH) stretching vibration are represented. Then, the peaks located at 2929 cm^−1^ and 3400 cm^−1^ correspond to n(CH_2_) and n(OH) stretching vibrations [20,21], respectively. The peak at about 3500 cm^−1^ is representative of –OH groups in the Na–CMC chemical structure and those due to the adsorbed moisture (Figure S1b in Supporting Information file). From Figure 1a it is evident that the spectrum of the composite and that of the pure PDMS are nearly the same. The diffraction patterns, shown in Figure 1b, highlight the presence of two different peaks for pure PDMS as confirmed in the literature [22]: the first one, exhibiting bigger amplitude, is located at around 11.65°, while the second one, smaller and broader, lies at 20.68°, thus suggesting an amorphous microstructure of the polymer [23]. Na–CMC exhibits a broad peak centered at 20°, representative of the low crystallinity degree of the Na–CMC structure. Therefore, the starting materials employed in this study are all defined as amorphous. In the structure of the composite material, the two peaks, at 11.65° and 20°, are both observed. The results of the thermal analysis are shown in Figure 1c,d, where TGA and DSC thermograms are respectively shown.

The TGA curve related to PDMS exhibits a main step, occurring between 330 °C and 630 °C and representative of the degradation of the polymer-silicone backbone. An initial, slight weight loss, corresponding to approximately 0.3% of the initial mass, is due to moisture content. After 630 °C, the residual mass is minimal, around 0.01%, indicating that decomposition products are volatile.

TGA signal of Na–CMC, shown in detail in Figure S2 (in the Supporting Information file), reveals four different degradation steps, explained by the fact that Na–CMC is a complex copolymer composed by different chemical groups. The first step, completed at about 250 °C and corresponding approximately to 12% of weight loss, is due to moisture evaporation; the second weight loss, the most considerable (around 62.5%), lies in the range 200–550 °C (inflection point at 289.13 °C) and is representative of the degradation of the saccharide rings, the breaking of C–O–C bonds in the CMC chain, and the elimination of CO_2_ from the polymeric backbone [24,25]; the third weight loss, around 21.5%, exhibits an inflection point at 697.66 °C and corresponds to the degradation of further organic material; while the last step, with a weight loss of about 11% and an inflection point at about 935.36 °C, is representative of sodium evaporation. In this case, a minimal residual mass is also proved in the end of the measurement, indicating that the decomposition products are volatile.

From Figure 1c it is evident that the presence of cellulose in the composite filament anticipates the beginning of the softening and degradation processes. It also modifies the rate of degradation of PDMS matrix, as denoted by the higher slope of the Na–CMC/PDMS curve when compared to the bare PDMS one. On the contrary, from Figure 1d it emerges that the cellulose presence in the composite does not cause significant modifications in the DSC thermogram. DSC thermograms of the pure starting materials, shown in Figure S3 (Supporting Information file), reveal that PDMS softens rather than melts, confirming its amorphous character, while Na–CMC appears to be a highly hygroscopic material.

**Figure 2:**
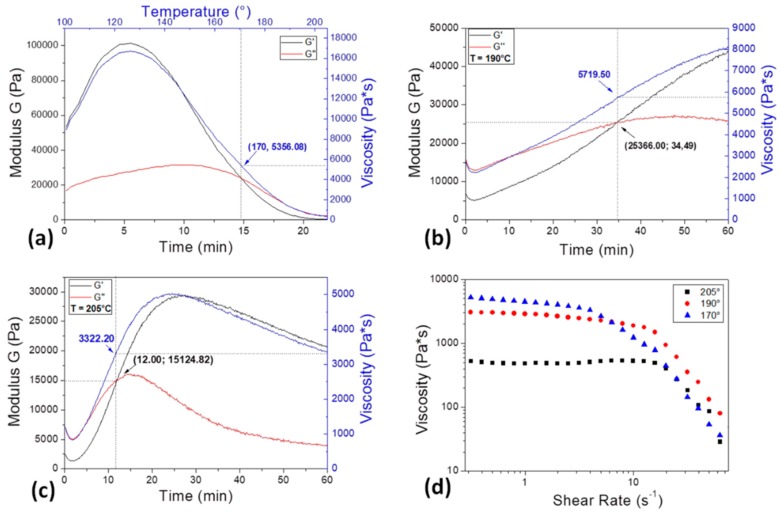
Rheological analysis on PDMS pellets: (**a**) dynamic rheological ramp test; (**b**) isothermal characterization at 190 °C and (**c**) at 205 °C; (**d**) viscosity vs. shear rate at different temperatures of interest.

In order to fix the working temperatures of the extruder chambers, used to shape PDMS pellets into filaments successively processed by the 3D printer, a preliminary rheological characterization has been carried out on PDMS pellets, as reported in Figure 2.

A dynamic rheological ramp test has been firstly performed in Figure 2a, where the trend of viscosity with respect to temperature variations and of G modules with respect to time have been evaluated, in order to assess the minimum processing temperature and a possible degradation of the material could suffer during the extrusion process. The test has been performed by varying temperature from 100 °C to 205 °C, according to the recommendation for extrusion in the polymer datasheet. The obtained results, shown in the graph, highlight a viscoelastic behavior of PDMS pellets. The intersection of storage and loss modules, G′ and G″, corresponding to the complete material softening, occurs at a temperature of about 170 °C, a value in agreement with the “melting range” reported in the material datasheet (170–205 °C). The viscosity increase, recorded in the beginning of the test, is most likely due to the thermal expansion to which the material is subjected and is a typical behavior of viscoelastic materials, like polymers. After its maximum, reached at 127 °C, viscosity starts to decrease as the material softens, and continues until the end of the test, when temperature reaches the value of 205 °C, the upper limit of the indicated “melting range”. After repeated rheological tests (data not shown) were performed to fix the working temperature of the different extruder chambers, two temperatures have been selected, 190 °C and 205 °C, at which rheological isothermal analysis has been then performed (Figure 2b,c). From the comparison of Figure 2b,c it emerges that by increasing the temperature, polymer softening occurs before, respectively after 34.49 min at 190 °C, and after 12 min when kept at 205 °C.

Figure 2d is representative of the viscosity as a function of the shear rate, at three specific temperature values: the obtained softening temperature of 170 °C, and those chosen as the extruder working temperatures, that are 190 °C and 205 °C. All the acquired curves exhibit the characteristic behavior of pseudoplastic materials, typical of many polymers in the melt/soften state, and, while increasing temperature, the viscosity values decrease, as expected.

### 3.2. Extrusion of PDMS and Na–CMC/PDMS Composite Filaments

Based on the results of the rheological tests and on recommendations in the extruder datasheet, the chosen extruder parameters are those summarized in Table 1, where the highlighted lines correspond to parameter values optimized through the performed rheological characterization.

**Figure 3:**
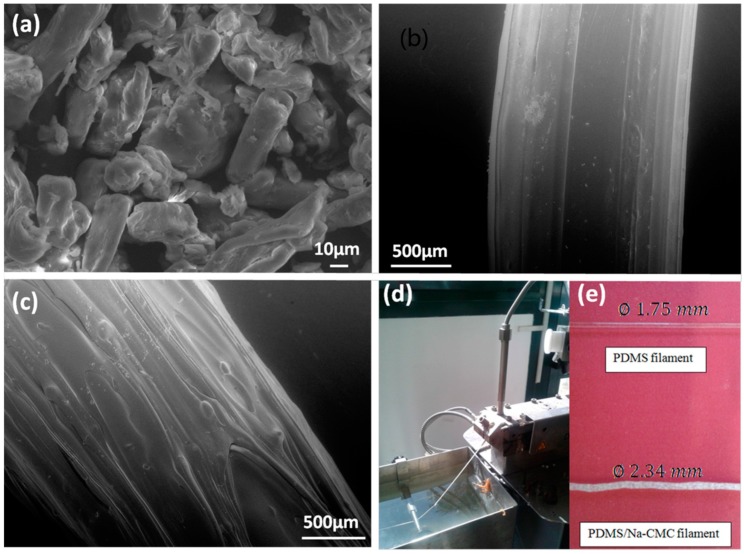
Scanning Electron Microscopy (SEM) images of the (**a**) employed Na–CMC powder; (**b**) bare PDMS filament surface; (**c**) Na–CMC/PDMS composite filament; photographs of (**d**) one filament during the extrusion process and (**e**) the two kinds of obtained filaments.

Once the starting materials have been deeply characterized and extruder parameters fixed, filaments of both pure PDMS and Na–CMC/PDMS composite have been fabricated as described in Materials and Methods Section 2 and shown in Figure 3, where SEM micrographs of the starting cellulose powder and of the two extruded filaments are shown. In Figure 3a it is possible to notice the characteristic fibril shape of cellulose particles, with a diameter of about 10 µm and a length of tens of microns. The presence of these fibrils is clearly evident from the comparison of Figure 3b,c, representing, respectively, the neat PDMS filament, and the Na–CMC/PDMS composite one. In Figure 3d–e, photos of the extrusion process and the bare PDMS and the produced Na–CMC/PDMS composite filaments, with their diameter size, can also be observed. It is possible to notice that, even though fixing the same extruder parameters, the composite filament always exhibits higher diameter value, equal to 2.34 mm. Moreover, the addition of cellulose-based filler into the polymeric matrix leads to obtain a filament that is not transparent and rougher than that made by pure PDMS, and also characterized by a slimy effect.

### 3.3. Rheological Properties of PDMS and Na–CMC/PDMS Filaments

A comparison between the two kinds of filaments rheological properties has been then carried out in order to investigate the influence of the cellulose addition on the PDMS processability in the further FDM step and assess the working parameters of the 3D printer.

**Figure 4:**
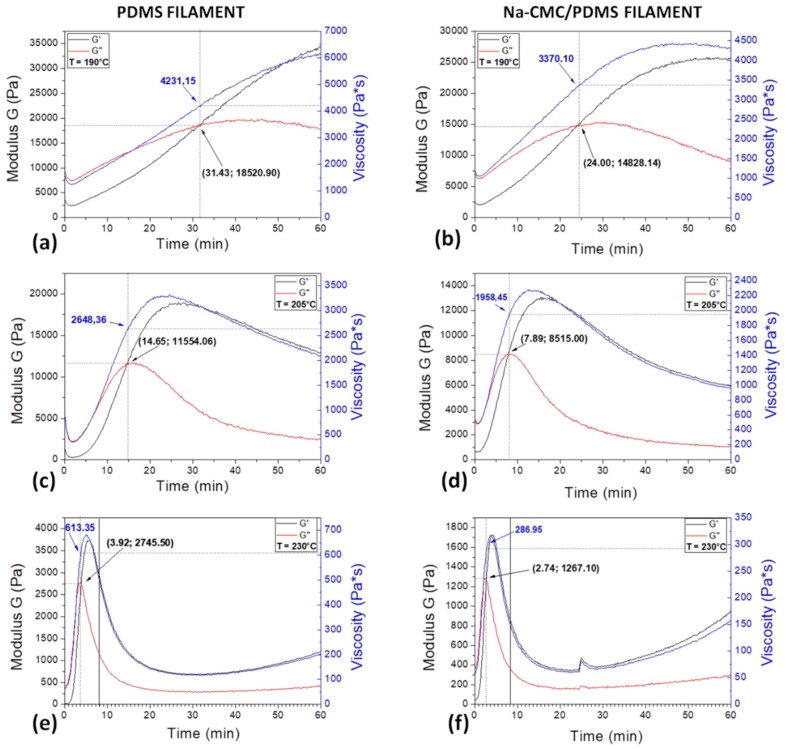
G′, G″ and viscosity curves as a function of time under varying process temperature, for both the (**a**,**c**,**e**) bare PDMS and (**b**,**d**,**f**) composite Na–CMC/PDMS filament.

First of all, isothermal analysis has been performed at 190 °C (Figure 4a,b) and 205 °C (Figure 4c,d), the same temperatures at which PDMS pellets were previously characterized and then extruded, as the already extruded filaments have to undergo to a further “ideal” extrusion process in the printer. Moreover, the same characterization has been performed at 230 °C (Figure 4e,f) that is the temperature that will be finally chosen for the 3D-printing step, as deeply explained in the Section 3.4 “Filament 3D printing”. The results are reported in Figure 4, where the graphs in the first column are referred to the neat PDMS filament, while those in the second one to the Na–CMC-modified PDMS filament. The graphs show G′ and G″ modules as a function of time and the viscosity curve recorded at the specified temperature. The crossover point, at the intersection between G′ and G″, and the corresponding viscosity values have been highlighted in each graph. In both filament cases, when temperature is increased, the related timespan time is reduced, as observed also in the case of pure pellets. Moreover, the addition of cellulose fibrils in the PDMS matrix moves up the occurrence of the material softening, as the inflection point lies at shorter time interval in graphs of Figure 4b,d, rather than in that of Figure 4a,c.

The main data of the described rheological analysis are summarized in Table 2.

From data summarized in Table 2 it can be deduced that, for a fixed temperature value, melting time decreases (the intersection of G′ and G″ modules occurs before) moving from PDMS pellets to PDMS filament. This is most probably due to the fact that PDMS filaments have been already thermally treated during the previous extrusion process in order to acquire their final shape. The presence of cellulose facilitates even more the silicone-melting process; indeed, in the case of the Na–CMC/PDMS filament, the intersection of modules occurs before those of PDMS pellets and filament. At the same time, moving from PDMS pellets to pure and composite PDMS filament, the modulus and viscosity values decrease. Therefore, the presence of cellulose causes a reduction in viscosity. Moreover, from Table 2 it emerges that, for each type of sample, a temperature increment causes the crossover point to move up and a reduction of the modulus and viscosity values.

**Figure 5:**
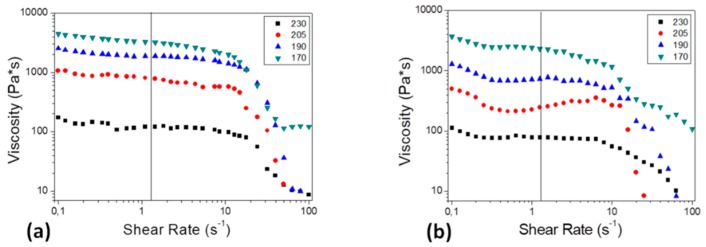
Viscosity vs. shear rate at different temperatures of interest for (**a**) neat PDMS and (**b**) composite Na–CMC/PDMS filaments.

Further rheological tests have been carried out on the filaments in order to characterize their behavior during the printing process, at the temperatures of interest, and results are shown in Figure 5.

Figure 5 is representative of the variation of viscosity when varying the shear rate for both PDMS and Na–CMC/PDMS filaments, in correspondence with the softening temperature found for PDMS pellets, the two selected for the extrusion process and the one that will result as the most suitable for the printing (170, 190, 205, and 230 °C, respectively). All the examined curves show the typical trend of a pseudoplastic material, typical of many polymers in the melt state.

The shear rate value at which filaments are subjected to during the 3D-printing process is calculated: for a typical 3D-printing speed of 2 mm/s and an obtained filament diameter of 1.6 mm, dependent on the nozzle size, a shear rate of 1.25 s^−1^ is calculated as their ratio. At this value, moving from lower to higher temperatures the viscosity decreases, and the same trend is evident also in the case of bare PDMS pellet viscosity (Figure 2d,e). Moreover, the composite filaments always exhibit lower viscosity values, as also shown in Figure 2e. Interestingly, in the case of the composite filament, the viscosity values at different shear rates and temperatures (in the range of 3D-printer working conditions) show the same trend of PLA-composite filaments, traditionally employed in FDM applications [26].

### 3.4. Filament 3D Printing

**Figure 6:**
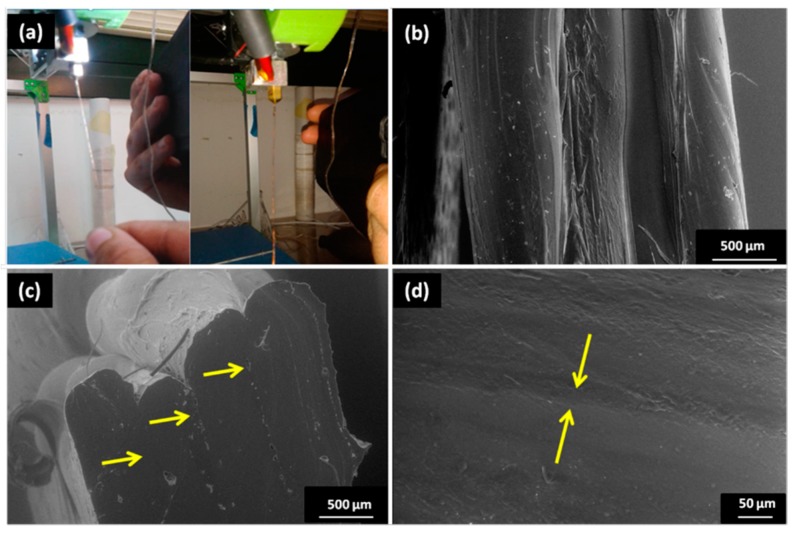
Photos of (**a**) the neat PDMS and composite Na–CMC/PDMS filaments used in FDM; SEM images, at different magnification, of (**b**–**d**) the composite filament printed in layers. Arrows indicate the interface between different layers.

Once the composite filament has been produced by extrusion and deeply characterized, also in comparison with the pure PDMS one, it is tested in FDM. Despite the numerous attempts of 3D printing at temperature values of 190 °C and 205 °C, and related time interval before material degradation, no satisfying results have been obtained. Indeed, the evident surface roughness, larger diameter, and the faster degradation of the composite filament caused nozzle obstruction, which prevented the printing process completely, or led to the printing of a degraded filament. Therefore, further combinations of temperature and related span time have been tested, and, among these, the one obtained setting a temperature of 230 °C, with a corresponding span time of 2.74 min (3.92 for the pure PDMS filament), even if too short, resulted to be effective in 3D printing; indeed, lower temperature values did not permit to print at all. In this way, a first attempt of FDM of the composite PDMS filament has been performed, as a primary step for the production of three-dimensional objects, such as a model of human heart. Figure 6 shows photos of the satisfying filament used for FDM (Figure 6a) and micrographs of their deposition in layers (Figure 6b–d), where it is possible to notice that layers adhere between each other completely, and the interfaces are pointed at by the arrows. Nevertheless, highly irregular samples have been obtained (see Figure S4), as the employed 3D printer did not permit a fine control of geometry while using the current material. This is most probably due to the intrinsic nature of the used polymeric matrix, which does not properly melt, but softens and degrades very quickly. On the other hand, PDMS-like polymers are often successfully 3D-printed by making changes in the traditional FDM experimental setup, for example, by switching the basic filament-dispensing system of an FDM machine with a syringe and needle, allowing them to avoid modifications in the stage, software, or the printing unit [27]. Hinton et al. [28] used a hydrophilic support bath via freeform reversible embedding to extrude PDMS within the hydrophilic Carbopol gel that is later cured by heating in two rounds, managing to create perfusable manifolds using this technique. Li and group printed wax instead of PDMS by replacing the syringe with a glass nozzle [29]. Subsequently, they use these wax prints as molds for casting PDMS. This approach results to be better than direct-printing PDMS, as it obviates the need of employing a complicated gel matrix-based printing, leading to smoother surfaces at the same time as wax reduces the process-introduced roughness.

Hence, when using a silicon-based polymer, also modified with a cellulose filler, the use of different and more robust 3D printers, and/or a modified printing process are strongly recommended.

## 4. Conclusions

The work presented and discussed so far represents a preliminary, feasibility study of the opportunity to use a low-cost FDM-dedicated instrumentation for printing a PDMS-based composite material, in order to obtain 3D models of human organs, like the heart, to be used for surgical and professional education applications. Indeed, the addition of Na–CMC fibrils as a filler succeeds in the goal of improving the material tactile properties, mimicking the slimy tactile effect of living organs. Both the neat PDMS and the composite Na–CMC/PDMS filaments are produced by extrusion. With respect to the neat PDMS filaments, the composite ones exhibit larger diameter and roughness that cause undesirable effects during 3D printing, such as episodic nozzle obstruction. Moreover, the composite filament exhibits faster degradation that contributes in preventing the printing of three-dimensional items with fine control of geometry. Although the cellulosic filler hinders the FDM step, the major issues are related to the low crystallinity degree of the employed polymeric matrix. The mentioned limitations could be overcome by employing a silicon-based polymer with a higher crystallinity degree, or by opting for FDM modifications that render 3D printing more suitable for PDMS-like polymers, like the ones mentioned. 

## Figures and Tables

**Table 1:** Employed extrusion parameters.

Extruder Parameters	Value
Screw speed (rpm)	15
Feeder speed (of screw)	10%
Feed zone temperature (°C)	100
Compression zone temperature (°C)	205
Metering zone temperature (°C)	205
Die temperature (°C)	190

**Table 2:** Synthesis of the main data obtained from the rheological analysis for PDMS pellets and filament and for Na–CMC-modified PDMS filament. Different gray scale is used to highlight different temperature values.

Sample Name	Temperature (°C)	Crossover Point		
		Time (min)	Modulus (Pa)	Viscosity (Pa·s)
**Pellets**	190	34.49	25,366.00	5719.50
**Pellets**	205	12.00	15,124.82	3322.20
**PDMS filament**	190	31.43	18,520.90	4231.15
**PDMS filament**	205	14.65	11,554.06	2648.36
**PDMS filament**	230	3.92	2745.50	613.35
**PDMS/Na–CMC filament**	190	24.00	14,828.14	3370.10
**PDMS/Na–CMC filament**	205	7.89	8515.00	1958.45
**PDMS/Na–CMC filament**	230	2.74	1267.10	286.95

## Supplementary Materials

The following are available online at http://www.mdpi.com/1996-1944/11/9/1578/s1. Figure S1: FT_IR characterization on PDMS and Na-CMC powder, Figure S2: TGA thermogram of Na-CMC powder, Figure S3: DSC thermogram of PDMS and Na-CMC powder, Figure S4: Photos of the 3d-printed samples.
