# Supplementary material for: A Feasibility Study of Processing Polydimethylsiloxane–Sodium Carboxymethylcellulose Composites by a Low-Cost Fused Deposition Modeling 3D Printer
Source: Materials (Basel). 2018 Sep 1;11(9):1578. doi: 10.3390/ma11091578 (PMC6163707; doi:10.3390/ma11091578)
Supplement: Supplementary file 1 [file materials-11-01578-s001.pdf]

Supporting Information file

A Feasibility Study of Processing Polydimethylsiloxane–Sodium Carboxymethylcellulose Composites by a Low-Cost Fused Deposition Modeling 3D Printer

Paola Calcagnile [1], Gabriele Cacciatore [1], Christian Demitri [1], Francesco Montagna [1], Carola Esposito Corcione [1]*

[1] Dipartimento di Ingegneria dell'Innovazione, Università del Salento, via Monteroni, Km 1, Lecce, 73100, Italy. paola.calcagnile@unisalento.it; gabriele88cacciatore@gmail.com; christian.demitri@unisalento.it; francesco.montagna@unisalento.it.

* Correspondence: carola.corcione@unisalento.it; tel.: +39-0832-297326

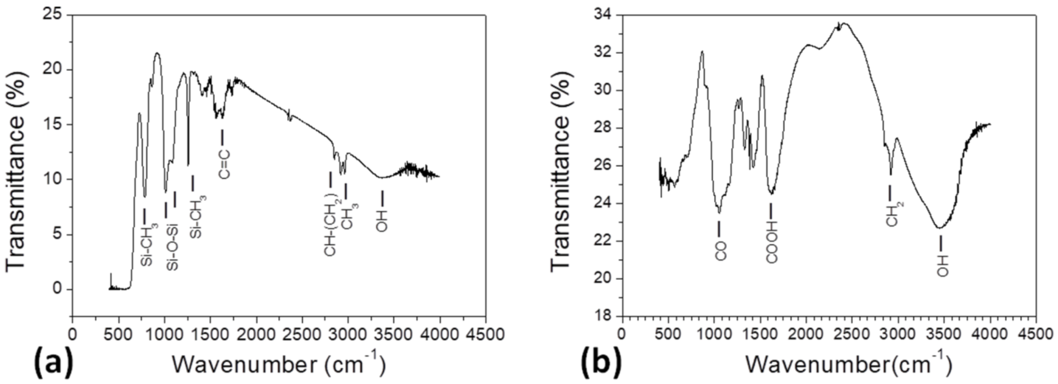

Figure S1. FTIR characterization performed on (**a**) PDMS and (**b**) Na–CMC powder, with specified typical peaks.

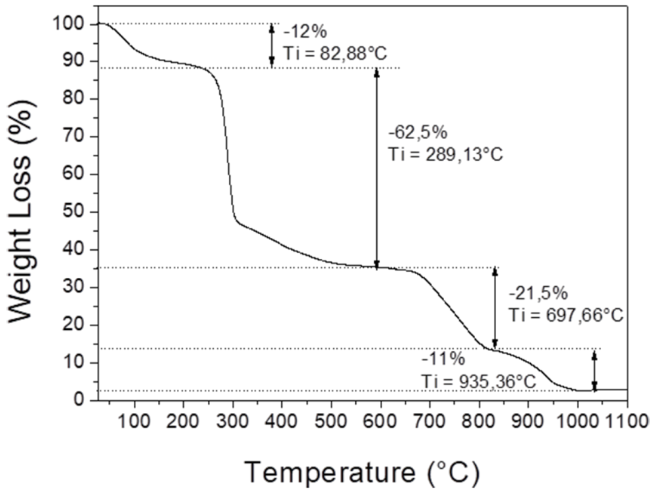

Figure S2. TGA thermogram of Na–CMC powder with highlighted weight loss steps.

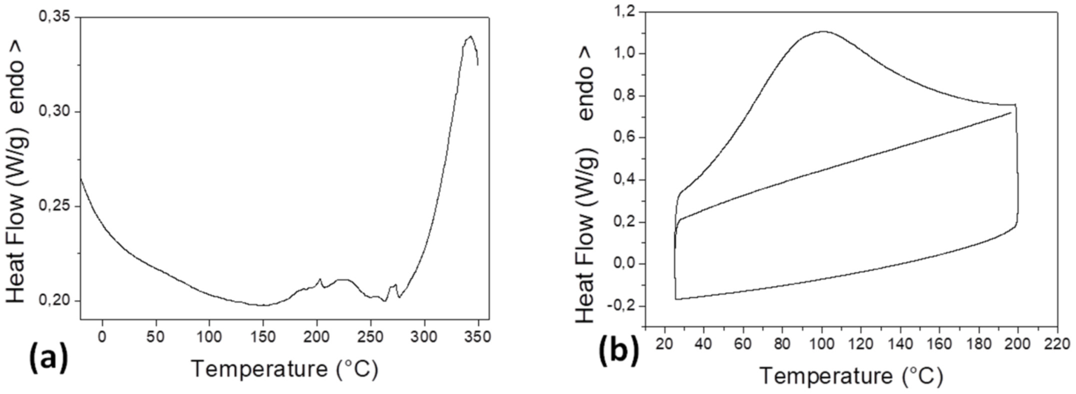

Figure S3. DSC thermogram of (**a**) PDMS and (**b**) Na–CMC powder.

DSC analysis in Figure S3a was performed on a Mettler-Toledo 822 (Mettler-Toledo, Greifensee, Switzerland) instrumentation in order to investigate the thermal behavior of the employed materials. For PDMS pellets, the analysis was performed under a nitrogen flux of 30 mL min^{-1} applying a heating scan between –25 and 350 °C at 10 °C min^{-1}.

DSC program in Figure S3b for Na–CMC powder is based on a heating scan between 25 and 200 °C at 10 °C min^{-1}, followed by a cooling one between 200 and 25 °C with a rate of 5 °C min^{-1} and a further heating scan equal to the first one.

In Figure S3a, DSC signal of PDMS, exhibiting a large concavity between 100 °C and 210 °C, suggests that the material softens rather than melting, confirming the amorphous character of the polymer microstructure. Moreover, the signal profile above 250 °C is representative of occurring polymer degradation, which reaches its maximum at about 330 °C.

DSC of Na–CMC in Figure S3b highlights that the material is highly hygroscopic and, until 200 °C, it is not subjected to any kind of modification. Indeed, in the first heating scan, DSC thermogram reveals a broad peak centered at about 100 °C, representative of the evaporation of a considerable amount of water, while no peaks are revealed in the second heating scan, performed after the cooling one.

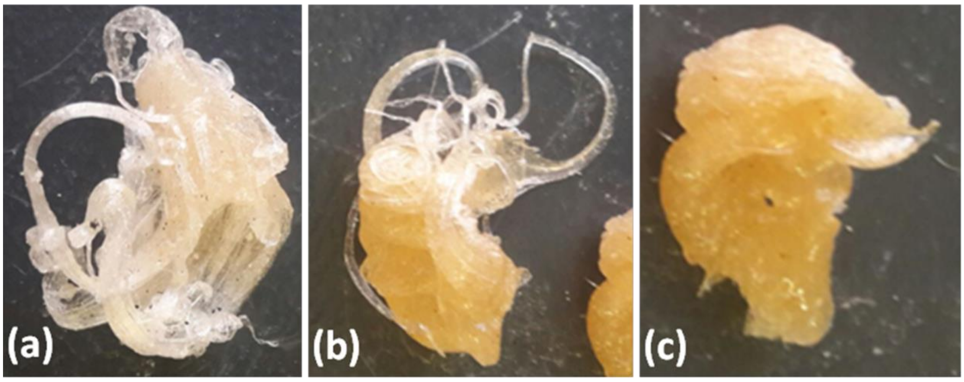

Figure S4. (**a**) (**b**) (**c**) Photos of the 3D-printed samples.
